# Chemistry of Fullerene Epoxides: Synthesis, Structure, and Nucleophilic Substitution-Addition Reactivity

**DOI:** 10.3390/molecules17066395

**Published:** 2012-05-25

**Authors:** Yusuke Tajima, Kazumasa Takeshi, Yasuo Shigemitsu, Youhei Numata

**Affiliations:** 1 Organic Optoelectronics Laboratory, RIKEN, 2-1 Hirosawa, Wako, Saitama 351-0198, Japan; 2 Graduate School of Science and Engineering, Saitama University, 255 Shimoohkubo, Saitama 338-8570, Japan; 3 FLOX Corporation, 2-3-13 Minami, Wako, Saitama 351-0104, Japan; 4 Photovoltaic Materials Unit, National Institute for Materials Science, 1-2-1 Sengen, Tsukuba, Ibaraki 305-0047, Japan

**Keywords:** fullerene, epoxidation, regioselectivity, nucleophilic substitution, Lewis acid

## Abstract

Fullerene epoxides, C_60_O_n_, having epoxide groups directly attached to the fullerene cage, constitute an interesting class of fullerene derivatives. In particular, the chemical transformations of fullerene epoxides are expected to play an important role in the development of functionalized fullerenes. This is because such transformations can readily afford a variety of mono- or polyfunctionalized fullerene derivatives while conserving the epoxy ring arrangement on the fullerene surface, as seen in representative regioisomeric fullerene polyepoxides. The first part of this review addresses the synthesis and structural characterization of fullerene epoxides. The formation of fullerene epoxides through different oxidation reactions is then explored. Adequate characterization of the isolated fullerene epoxides was achieved by concerted use of NMR and LC-MS techniques. The second part of this review addresses the substitution of fullerene epoxides in the presence of a Lewis acid catalyst. Most major substitution products have been isolated as pure compounds and their structures established through spectroscopic methods. The correlation between the structure of the substitution product and the oxygenation pattern of the starting materials allows elucidation of the mechanistic features of this transformation. This approach promises to lead to rigorous regioselective production of various fullerene derivatives for a wide range of applications.

## 1. Introduction

Since the first detection of fullerene epoxides via mass spectrometry in a fullerene mixture generated by the arc discharge of graphite in 1991, many studies on fullerene oxides have been performed for the purpose of developing new materials. The epoxidation of fullerene can proceed readily in the presence of oxidants such as ozone [1], organic peroxide [2], dimethyldioxirane [3], methyltrioxorhenium-hydrogen peroxide [4], and cytochrome P450 [5]. Under several circumstances, in different oxidations fullerene C_60_ has been shown to give higher oxides (C_60_O_n_, n ≥ 2) that possess only a few regioisomers. For instance, although there are indeed eight possible regioisomers of C_60_O_2_ and 43 isomers of C_60_O_3_ given the multiple reaction sites available on the C_60_ cage [6], the actual products of most oxidations are only two isomers of C_60_O_2_ and three isomers of C_60_O_3_. However, few C_60_O_n_ isomers have been isolated and identified thus far, and experimental data on the regioselective epoxidation of C_60_ are also scarce.

Meanwhile, the structures of C_60_O_n_ isomers were first studied theoretically on the basis of the thermodynamic stability of ground state molecules and on the dynamic behavior of the molecules via the transition states. Those results regarding the structures of C_60_O and the predominant isomers of C_60_O_2_ can explain these experimental observations. Manoharan showed computationally that multiple epoxidations of C_60_ preferentially proceeds at the adjacent rather than distant double bonds of the existing epoxide group, and predicted that multiple epoxidations should occur on one benzenoid ring of C_60_ to form the C_60_O_3_ isomer with *C*_3v_ symmetry. Feng *et al*. studied three isomers of C_60_O_3_ by using the semiempirical quantum mechanical INDO method, and predicted that the three types of C_60_O_3_ isomers with *C*_3v_, *C*_s_ and *C*_2_ symmetries, respectively, should be very stable, and reported their calculated electronic spectra [7]. Curry *et al*. predicted that the three lowest-energy isomers (*C*_3v_, *C*_s_ and *C*_2_) of C_60_O_3_ should exist in equilibrium at room temperature by using a modified and extended Hűckel method [8].

Previously, we found three types of C_60_O_3_ isomers in a reaction solution of C_60_ with *m*-CPBA by means of a chromatographic technique involving the use of two different columns [9]. Electronic spectroscopy and mass-spectroscopy examinations of these isomers were mostly consistent with the calculated results by Feng *et al*. However, the precise structures of these isomers could not be confirmed by ^13^C-NMR and X-ray studies, which are unavailable due to the low solubility and poor crystallinity. We carried out measurements of FT-IR and ^13^C-NMR spectra precisely after the isolation and further purification of two types of C_60_O_2_ isomers and three types of C_60_O_3_ isomers. Simultaneously, we demonstrated experimentally the regioselectivity of the epoxidation of C_60_ by means of the identification of products from each oxidation of the isolated isomers. 

Fullerene epoxides exhibit interesting properties applicable to new materials development. However, the chemical transformations of fullerene epoxides have been studied sparingly, despite the general recognition that they could serve as convenient starting materials for the synthesis of functionalized fullerene derivatives [10]. We first succeeded in converting fullerene epoxide stoichiometrically into a 1,3-dioxolane derivative [11]. Reaction of C_60_O with benzaldehyde in the presence of a Lewis acid led to the formation of a 1,3-dioxolane derivative of C_60_ in high yield. This implies the possibility of other nucleophilic substitutions of the epoxy rings on a fullerene cage. The chemical transformation of fullerene epoxide is expected to play an important role in the development of functionalized fullerenes, because such transformations can readily afford a variety of mono- or polyfunctionalized fullerene derivatives, such as regioisomeric fullerene polyepoxides, while conserving the epoxy ring arrangement on the fullerene surface. The recent development of large-scale production techniques for fullerene epoxide [12] thus prompted us to develop a new methodology to synthesize polyfunctionalized fullerene derivatives by means of efficient chemical transformation of regioisomerically pure fullerene polyepoxides. Then, we also achieved the efficient formation of fullerene derivatives from C_60_O with aromatic nucleophilic compounds by Lewis acid-assisted nucleophilic substitution of the epoxy ring. The direct substitution of epoxide oxygen atoms on the fullerene epoxide—a versatile and advantageous synthetic methodology we report here—provides highly regioselective access to a variety of fullerene adducts.

## 2. Epoxidation of Fullerene C_60_ and Their Regioisomeric Structure

**Figure 1:**
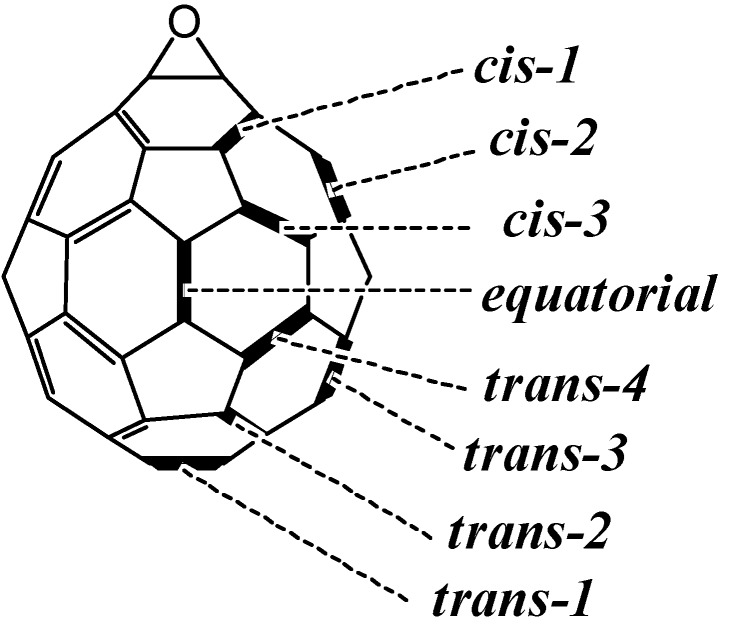
Positional notation of the fullerene epoxide, C_60_O.

**Figure 2:**
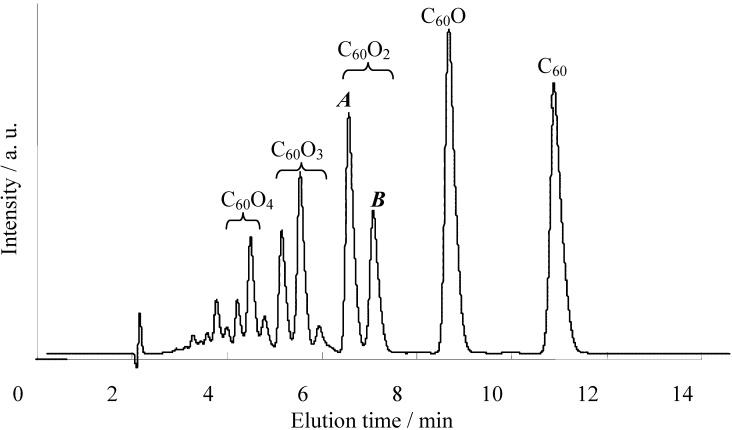
HPLC reversible-phase chromatograms (Develosil C30 RPFULLERENE, 34:66 acetonitrile/toluene, 335 nm detection) for reaction mixtures resulting from the *m*-CPBA of C_60_ (in toluene, 80 °C, 30 min).

The epoxidation of C_60_ generally affords the higher fullerene epoxides (C_60_O_n_, n ≥ 2), which have only a few types of regioisomers. For instance, the epoxidation of C_60_ with *m*-chloroperoxybenzoic acid (*m*-CPBA) forms a few C_60_O_2_ regioisomers (see Figure 1 for positional notation of C_60_O) that can be separated into two fractions by high-performance liquid chromatography (HPLC) (peaks ***A*** or ***B*** in Figure 2), although from the standpoint of the multiple reacting sites available on a C_60_ cage there are eight possible regioisomers of C_60_O_2_ [6]. A major C_60_O_2_ fraction is known to be composed of only one isomer with both oxygen atoms positioned over 6:6 ring junctions on a common six-membered ring of the carbon cage, namely the *cis-1* adduct [5]. Meanwhile, a minor fraction of C_60_O_2_ has been considered to contain more than one isomer [13]. These isomers could not be separated by any HPLC column and their precise structures have hardly been ascertained by ^13^C-NMR or X-ray studies because of the low yield and the non-crystalline nature of the products.

The splitting pattern of each fraction in HPLC varies with both the position of the epoxy group on the C_60_ cage and its structural symmetry. A detailed comparison of these patterns is informative in relation to the structure of existing C_60_O_2_ isomers. In order to explain the isomeric structure of the preferentially formed C_60_O_2_, we also performed calculation of the static reaction index of oxygen addition sites on the C_60_ cage, and compared the experimental observations with the calculated results [14].

In the past, the structure of C_60_O_n_ isomers was studied on the basis of the thermodynamic stability of ground state molecules and the dynamic behavior of the molecules via the transition states. The results of these studies can explain the experimental observations regarding the structures of C_60_O and the predominant isomer of C_60_O_2_. Manoharan demonstrated computationally that the multiple epoxidations of C_60_ preferentially proceeds at the adjacent rather than distant double bonds of the existing epoxide group [15]. By using a modified extended Hűckel method, Curry *et al*. also correctly predicted for C_60_O_2_ that the *cis-1* adduct has exceptionally low energy compared to the next stable isomers [8]. Feng *et al*. studied the possible structure of C_60_O_2_ isomers by using the semiempirical quantum mechanical INDO method, and showed that three types of C_60_O_2_ with the regioisomeric structures of *cis-1*, *cis-2*, and *trans-4* adduct, respectively, should be very stable [7]. A more detailed theoretical elucidation of the regioselective epoxidation on fullerene, however, is required to provide information concerning the possible structures of other experimentally obtained C_60_O_2_ isomers.

The reactivity for regioselective epoxidation on fullerene is assumed to be predicted by calculating the static reaction index for addition sites. On the basis of theoretical studies on the epoxidation of alkenes with peroxide [16,17], we hypothesized that the reaction index should fall between fullerene and *m*-CPBA. The electrophilicity of peroxide was attributed to its relatively weak O-O bond, which can provide an empty σ* orbital that can mix with the nucleophilic π-bond of fullerene. The electrophilic oxygen tends to attack the bonds with a high electron density. In the same manner, the epoxidation of fullerene by the electrophilic oxygen of *m*-CPBA is likely to occur on electron rich double bonds. Thus the electron density of a bond is expected to give a good index of a probable reaction site. A similar calculation has been reported for the first oxygen addition sites on fullerenes [18].

Since the epoxidation of fullerene is a step-wise reaction, the yield for C_60_O_n_ (n ≥ 2) can be calculated by considering the yield of the parent isomer C_60_O_n−1_. The electron density of a bond has been determined by semiempirical molecular orbital calculations (PM3). The yield of the *j*-th isomer of C_60_O_n_, *Y*_(_*_n,j_*_)_, can then be determined by the summation of the product of the yield of *i*-C_60_O_n−1_, *Y*_(*n*−1*,i*)_, and the branching probability from *i*-C_60_O_n-1_ to *j*-C_60_O_n_, *p_(i,j)_* as follows:


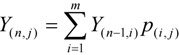


where *m* denotes the number of the parent isomer. If the branching probability increases linearly with the difference in electron density, *p_(__i,j__)_* is defined by utilizing the difference between the electron density of the bond of the addition site and the minimum electron density of the bond that undergoes epoxidation as follows: 


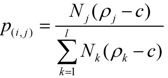


where *ρ_j_* and *N_j_* denotes the electron density of the bond of the addition site *j*, and the number of the symmetrically equivalent bond of *j*, respectively. The constant *c* is the minimum electron density that undergoes epoxidation. The variables *l* denotes the number of equivalent bonds of the possible addition sites.

**Figure 3:**
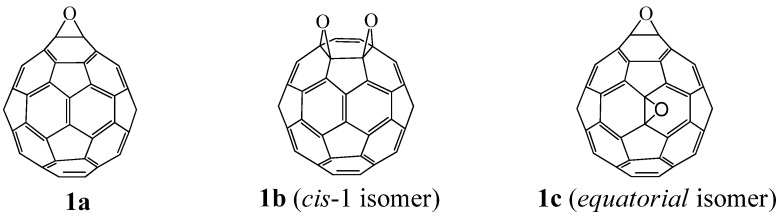
Fullerene mono-epoxide and regioisomeric di-epoxides isolated by means of preparative HPLC from a mixture of C_60_(O)_n_.

Table 1 shows the calculated yield for the C_60_O_2_ isomers, and the candidates for the dominant isomers based on the calculated yield. The *cis-1* adduct overwhelmingly dominated the other isomers. The next dominant isomer of C_60_O_2_ was predicted to be the *equatorial* adduct. The structure of the predominant isomer, the *cis-1* adduct (**1b** in Figure 3), agreed with the structure actually confirmed by X-ray analysis [2], whereas the next dominant isomer is differed from a previous suggestion [1] that the C_60_O_2 _isomers in fraction ***B*** are probably the *cis-2* or *trans-4* adducts. From both our experiment and calculations, at a minimum, we expect that the main product in fraction ***B*** is not the *trans-1* but rather the *equatorial* adduct of C_60_O_2_ (**1c** in Figure 3).

**Table 1:** Calculated percentage yield and point group of constitutional isomers of diepoxidized fullerene C_60_O_2_.

Structure	Symmetry	Yield / %
*cis-1*	*C*_s_	88
*equatorial*	*C*_s_	12
*trans-4*	*C*_s_	<0
*trans-2*	*C*_2_	<0
*trans-1*	*D*_2h_	<0

## 3. Lewis Acid-Assisted Reaction of Fullerene Epoxides with Nucleophiles

**Scheme 1:**
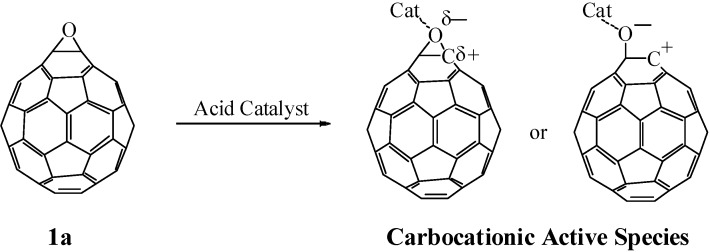
Generation of active carbocationic species from **1a** in the presence of an acid catalyst.

Epoxides are well-known to undergo heterolytic C–O bond cleavage in the presence of an acid catalyst to generate active carbocationic species for further reactions with nucleophiles. These active species react with various types of nucleophile such as carbonyl compounds, alcohols, and amines to afford 1,3-dioxolanes, 1,2-hydroxyethers, and 1,2-aminoalcohols, respectively. We have studied the reaction of fullerene epoxide **1a** with various nucleophiles in the presence of a Lewis acid. Reaction with a carbonyl compound led to the formation of a 1,3-dioxolane derivative in high yield; interestingly, reaction with a phenol or aniline derivative afforded an O- or N-heterocycle-fused fullerene derivative. These reactions are considered to proceed via a nucleophilic reaction on the carbocationic active species generated on the carbon atom of fullerene epoxide **1a** (Scheme 1). The formation of the heterocycle-fused fullerene derivative can be accounted for by the ring closure reaction induced by intramolecular dehydration of the initially formed 1,2-substituted product. In contrast, a Lewis acid-assisted nucleophilic reaction of C_60_O with an aromatic nucleophile such as toluene gave a 1,4-bisadduct. Application of these reactions to a regioisomeric fullerene polyepoxide would provide a convenient synthetic route to a variety of polyfunctionalized fullerene derivatives with a regioisomerically pure structure. These chemical transformations of fullerene epoxides are expected to play an important role in the development of functionalized fullerene cages. In this section, we review our recent progress in the work on the acid-assisted reaction of fullerene epoxides with various types of nucleophile.

### 3.1. Acetalization of Fullerene Epoxides

Several methods have been reported for the preparation of fullerene-fused 1,3-dioxolane derivatives, such as the reactions of C_60_ fullerene with dioxiranes [3], acyl hypohalogenites [19], and diacyl peroxides [20], or the reaction of C_60_ fullerene with a sodium alkoxide in the presence of air [21]. However, all of these reactions afforded fullerene-fused 1,3-dioxolane derivatives in only low to moderate yields, (*i.e*., <50%). As described in the previous section, we recently developed a preparative HPLC isolation method for some regioisomeric fullerene epoxides (Figure 3) [7]. Isolation of these epoxides prompted us to employ them as a starting material for efficient synthesis of fullerene-fused 1,3-dioxolane derivatives. We have found that the acetalization reactions of fullerene epoxide **1a** with various carbonyl compounds occur in the presence of a Lewis acid catalyst, an ion-exchange resin such as Amberlite, and a clay mineral such as montmorillonite to afford the corresponding 1,3-dioxolane derivatives in very high yields (Table 2) [9,22]. 

**Table 2:** Acetalization of fullerene epoxide **1a** with carbonyl compound in the presence of an acid catalyst.

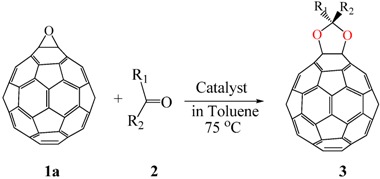				
Entry	2	Catalyst (equiv./amount)	Reaction Time	Yield ^a^ of 3 / %
1	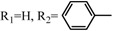	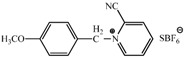 **4a **(0.29)	60 min	92
2	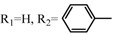	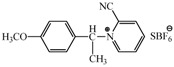 **4b** (0.28)	90 min	91
3	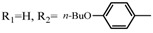	**4a** (0.29)	60 min	95
4	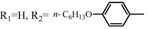	**4b** (0.28)	4.5 h	88
5	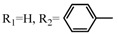	BF_3_·Et_2_O (one drop)	60 min	89
6	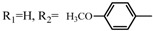	Amberlyst 15^® ^(268 mg/0.02 mmol)	3 h	96
7	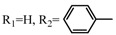	Montmorillonite (250 mg/0.02 mmol)	4 h reflux	60
8	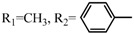	**4a** (0.27)	30 min	45
9	R_1_=CH_3_, R_2_=C_2_H_5_	**4a** (0.27)	30 min	44
10	Cyclohexanone	Amberlyst 15^®^ (250 mg/0.02 mmol)	65 °C, 5 h in benzene	60
11	γ-Butyrolactone	BF_3_·Et_2_O (one drop)	3 h in benzene	75

^a^ Isolated yield.

The reaction of a toluene solution of fullerene epoxide **1a** with excess amounts (200 equiv.) of a benzaldehyde derivative at 75 °C in the presence of a Lewis acid catalyst led to the formation of 1,3-dioxolane **3** in very high yield. Table 2 also shows that a similar acetalization of **1a** with other ketone compounds such as acetophenone, methyl ethyl ketone or cyclohexanone took place to give the corresponding 1,3-dioxolanes in moderate yields. Furthermore, a similar reaction of **1a** with γ-butyrolactone in benzene afforded an ortho-ester type 1,3-dioxolane derivative in high yield, although in toluene, it resulted in the formation of a 1,4-addition product of toluene to **1a**. This fact suggests that the nucleophilicity of a carbonyl group of an ester compound is not as strong as that of an aldehyde or a ketone, such that toluene instead acts preferentially as a nucleophile to the carbocationic species to yield a 1,4-addition product (1,4-bis(p-tolyl)-1,4-dihydro[60]fullerene). The Lewis acid catalyzed 1,4-addition reaction of aromatic nucleophiles to **1a** will be discussed in Section 3.2*.* Recently Gan and Zhang *et al*. reported that a Lewis acid such as BF_3_·Et_2_O catalyzes the acetalization reaction of the fullerene epoxide bearing *t-*butylperoxo groups to yield the corresponding 1,3-dioxolane derivatives in moderate yields [23].

**Scheme 2:**
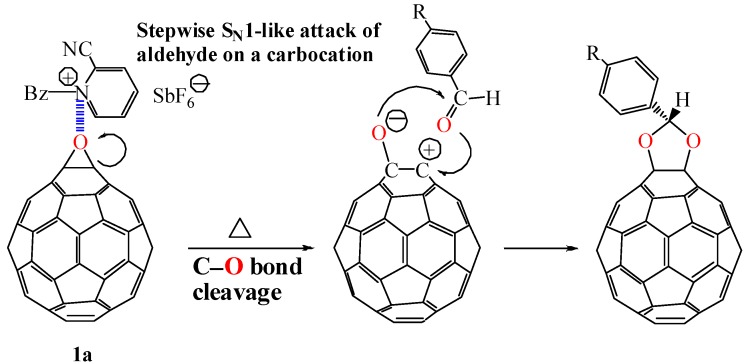
Stepwise S_N_1-like acetalization of fullerene epoxide **1a** with benzaldehyde derivative.

An epoxy ring is known to undergo a Lewis acid catalyzed acetalization to afford 1,3-dioxolane [24,25,26]. Generally the acetalization is assumed to follow a concerted S_N_2-like mechanism involving backside attack of a carbonyl oxygen on an epoxide carbon followed by rotation of the C–C bond of the epoxide and then the second C–O bond formation to produce a 1,3-dioxolane [26]. However, such a backside attack on a fullerene epoxide is not expected to occur owing to occupation of the entire side opposite the epoxy ring by the fullerene cage. According to this view, an acetalization of a fullerene epoxide is not expected to occur unless an alternative mechanism is involved in a front side attack of a carbonyl compound on the epoxy ring of the fullerene epoxide. The acetalization reaction of fullerene epoxide **1a** with benzaldehyde at 75 °C for 1 d did not proceed at all in the absence of a Lewis acid and resulted in the complete recovery of **1a**. This result suggests that the Lewis acid catalyzes the acetalization reaction to proceed by a stepwise S_N_1-like mechanism (Scheme 2). A Lewis acid induces a catalytic C–O bond cleavage of the epoxy ring to generate a carbocation on the carbon atom of the fullerene moiety. This is followed by nucleophilic attack of a carbonyl compound on the carbocation. An energetically unfavorable S_N_1-like attack of a carbonyl compound on the carbocation from the fullerene surface would be forced to occur with the requirement of relatively high activation energy for the C–O bond cleavage of the epoxy ring.

The activation energy for the reaction of **1a** with benzaldehyde was determined to be 112.7 kJ·mol^−1^ from the Arrhenius plots of ln k *vs*. 1/T, where k is the pseudo-first order rate constant and T is the reaction temperature. Based on the activation energy determined, the rate constant at room temperature (293 K) can be calculated to be 9.5 × 10^−7^ s^−1^, which is more than 10^3^ times smaller than that at 75 °C (k = 1.43 × 10^−3^ s^−1^). Therefore, that the Lewis acid catalyzed acetalization of **1a** with benzaldehyde did not occur at room temperature is unsurprising, although a similar acetalization of oxiranes such as but-2-ene epoxide or styrene oxide readily proceeds at room temperature and even at 0 °C to yield 1,3-dioxolanes according to an energetically favorable S_N_2-like mechanism [24,25,26].

The application of the above acetalization reaction to the regioisomerically pure fullerene di-epoxide **1b** (*cis*-isomer) or **1c** (*equatorial* isomer) would lead to chemical transformation of the two epoxy rings while conserving their arrangement on the fullerene cage. Under reaction conditions similar to those for mono-epoxide **1a**, the fullerene di-epoxides **1b** and **1c** were also subjected to acetalization with benzaldehyde derivatives to give bis-1,3-dioxolanes **5a** and **5b**, respectivelyinhigh yields (Table 3) [9,21]. The visible absorption spectra of **5a** and **5b** show the bands characteristic to those of the di-epoxides **1b** and **1c**, respectively, that is, **5a** showed a sharp band at 423 nm, which is the characteristic band for the *cis*-isomer **1b** [11,15]. This fact suggests that during the acetalization reaction, no migration of the carbocation on the fullerene moiety occurs and that the acetalization reaction of the fullerene di-epoxide proceeds while the rearrangement of the two epoxy rings on the fullerene cage is conserved.

**Table 3:** Acetalization of fullerene epoxide **1b** or **1c** with benzaldehyde derivatives **2** in the presence of pyridinium salt **4a** or **4b** shown in Table 2.

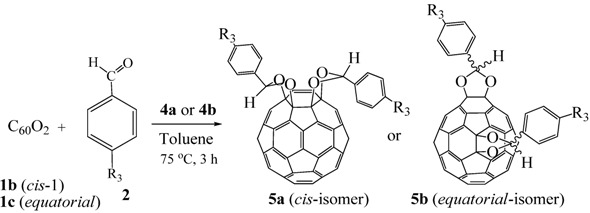			
Epoxide	2	Catalyst (equiv.)	Yield of 5 / %
**1b**	R_3_ = H	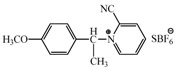 **4b** (0.28)	**5a** (R_3_ = H), 92
**1b**	R_3_ = *n*-BuO	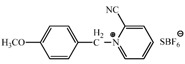 4a (0.29)	**5a** (R_3_ = *n*-BuO), 91
**1c**	R_3_ = H	**4b** (0.28 equiv.)	**5b** (R_3_ = H), 76
**1c**	R_3_ = *n*-BuO	**4a** (0.27 equiv.)	**5b** (R_3_ = *n*-BuO), 73
**1c**	R_3_ = *n*-C_3_H_7_	**4a** (0.27 equiv.)	**5b** (R_3_ = *n*-C_3_H_7_), 82

The ^1^H-NMR spectrum of **5a** (R_3_ = *n*-BuO) showed the two unequivalent acetal methine protons of equal intensity. In addition, it showed two sets of four phenyl protons and nine *n*-butoxy protons, also of equal intensity. Thus, the ^1^H-NMR spectrum of **5a** clearly demonstrates that the two phenyl groups are unsymmetrically disposed with respect to the fullerene moiety. The ^13^C-NMR spectrum of **5a** showed 51 signals for the fullerene sp^2^ carbons, indicating a lack of symmetry of the fullerene cage. Based on the above NMR analysis, *bis*-1,3-dioxolane **5a** is confirmed to have the structure of the stereoisomer depicted in Table 3. However, the configuration of the two phenyl groups in **5b** remains undetermined because no definitive information suggesting their configuration could be obtained from the ^1^H-NMR spectra of **5b**. We assume that **5b** is likely to be a mixture of two stereoisomers.

The fullerene di-epoxide **1b** (*cis*-isomer) or **1c** (*equatorial* isomer) also reacts with a ketone compound such as cyclohexanone in the presence of a Lewis acid catalyst or Amberlyst 15^®^ to yield the corresponding *bis*-1,3-dioxolane derivative in moderate yields (50–70%) [27]. These acetalization reactions are presumed to proceed with conservation of their arrangement on the fullerene cage.

The present work has clearly shown that the epoxy moiety of fullerene epoxides can be readily converted to 1,3-dioxolane derivatives in high yield by treatment with a carbonyl compound in the presence of a Lewis acid, ion-exchange resin or clay mineral. The acetalization reaction proceeds by a stepwise S_N_1-like mechanism which involves the nucleophilic attack of a carbonyl moiety at the carbocationic active species generated on the carbon atom of fullerene epoxides. Furthermore, the efficient bis-acetalization of a regioisomerically pure fullerene di-epoxide occurs under conservation of the arrangement of the two epoxy moieties to yield bis-1,3-dioxolane derivatives in high yield. These results could lead to development of a new methodology for facile synthesis of a variety of regioisomeric polyfunctionalized fullerene derivatives by means of efficient chemical transformation of a specific regioisomeric fullerene polyepoxide. In order to elucidate the reactivity of the carbocationic active species generated from fullerene epoxides, the next part of this section is oriented toward studies on the reaction of fullerene epoxides with nucleophiles other than carbonyl compounds.

### 3.2. Nucleophilic Substitution of Fullerene Epoxide with Aromatic Compounds

The reaction of a toluene solution of fullerene epoxide **1a** in the presence of 5 equiv. of boron trifluoride etherate (BF_3_·OEt_2_) at r.t. for 60 min led to the formation of 1,4-bis(*p*-tolyl)-1,4-dihydro [60]fullerene (**7a**) in 80.8% yield together with a very small amount of pristine fullerene C_60_ based on HPLC analysis [28]. Compound **7a** was purified by flash column chromatography on a silica gel with n-hexane as an eluent. A detailed spectral characterization of **7a** revealed that two *p*-tolyl moieties were introduced to the C_60_ core in a 1,4-addition pattern. The FT-IR spectrum was in good agreement with that in an earlier report [29,30]. The characteristic vibrations for the 1,4-adduct were found at 1,430, 1,187, 573, and 527 cm^−1^, and the C-H vibrations for toluene showed strong vibrations at 2,963 and 810 cm^−1^. The atmospheric pressure photochemical ionization (APPI) mass spectrum showed a molecular ion peak at *m/z* 902 (*i.e*., 166 units more than **1a**), corresponding to a 1:2 adduct of C_60_ and toluene-H. In the ^1^H-NMR spectrum, in addition to two doublets at 7.94 (2H), 7.93 (2H), 7.28 (2H), and 7.27 (2H) ppm for eight phenyl protons, one singlet methyl proton was observed at 2.46 (6H) ppm. The ^13^C-NMR showed the characteristic absorption of aromatic carbons in the *p*-tolyl group at 138.25 (2C), 136.91 (2C), 129.52 (4C), and 126.94 (4C) ppm, and two quaternary carbons of the C_60_ moiety were observed at 60.86(2C) ppm. The UV-VIS spectrum showed a characteristic absorption band around 450 nm. The 1,4-bisadduct structure for **7a** was evidently confirmed by comparing these spectra with FT-IR, ^1^H-NMR and ^13^C-NMR spectra for similar compounds [29,30].

Reactions of **1** with other aromatic compounds were carried out under the same conditions as described above, the results of which are listed in Table 4. In anisole, *o*-xylene and *m*-xylene the reaction produced the corresponding 1,4-bisadducts (**7b**, **7c** and **7d**) in 45–95% yields, whereas neither the 1,2-(**6e**) nor 1,4-bisadduct (**7e**) was produced in *p*-xylene. 

**Table 4:** Substitution of **1** with aromatic compounds.

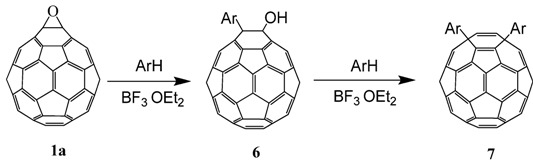			
	Aromatic Compound *^a^*	Isolated Yield of 6 / % *^b^*	Isolated Yield of 7 / % *^c^*
**a**	toluene	0	80.8
**b**	anisole	0	95.8
**c**	*o*-xylene	0	76.5
**d**	*m*-xylene	Trace	45.3 *^d^*
**e**	*p*-xylene	0	0
**f**	1,3,5-trimethylbenzene	35.3	Trace
**g**	chlorobenzene	0	0
**h**	benzene	0	0

*^a^* All reactions were performed according to the procedure described in the text; *^b,c^* All reactions were continued until complete disappearance of the starting material **1**; *^d^* Overall yield of the structural isomeric mixture of 1,4-adducts.

**Scheme 3:**
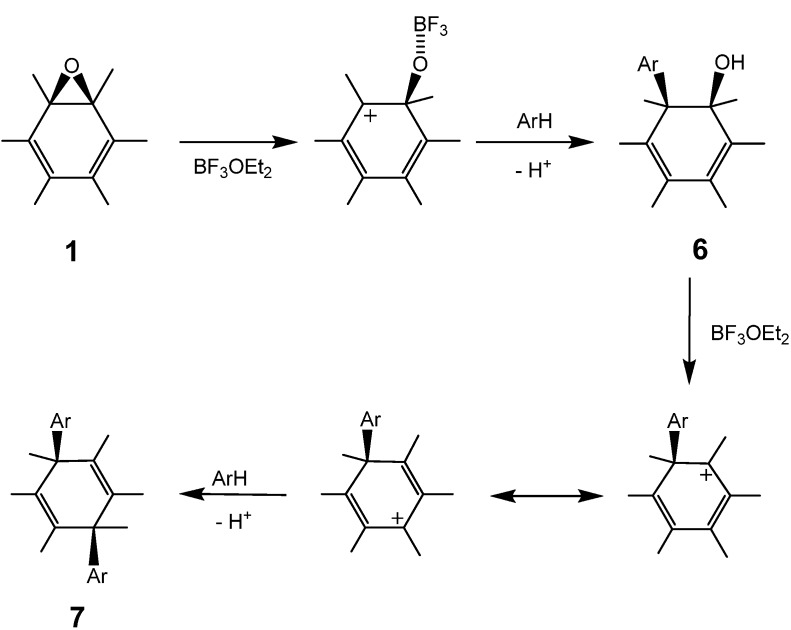
Proposed mechanism for the substitution of C_60_O with aromatic compounds. Part of the fullerene surface is shown.

Although the purification of 1,4-bisadducts **7a**, **7b**, and **7c** was easily carried out with the HPLC apparatus, **3d** was not well separated owing to the formation of structural isomers. In the case of 1,3,5-trimethylbenzene, the corresponding 1-(substituted phenyl)-2-hydroxyl-1,2-dihydro[60]fullerene (**6f**) was dominantly formed instead of the 1,4-bisadduct, as identified by UV-VIS, APPI mass, ^1^H- and ^13^C-NMR spectra. Usually the sp^3^ carbons of the C_60_ cage were observed to shift downfield *ca*. 10 ppm for 1,2-adducts relative to the 1,4-adducts. The two peaks at 86.9 and 72.0 ppm for the two sp^3^ carbons of the fullerene core in **6f** indicate a typical structure of 1,2-adduct. This suggests that the formation of 1,4-bisadduct from **1a** proceeds stepwise via 1,2-adduct **6** as an intermediate. In fact, the formation of **6d** can be observed as a transient intermediate in the course of the reaction in *m*-xylene. With respect to the UV-VIS spectrum beyond wavelengths of 300 nm, **6d** is identical to **6f**. The substitution reaction scarcely proceeded in benzene and chlorobenzene, even above 75 °C, and pristine C_60_ was observed instead of 1,4-adducts. The formation of pristine C_60_ without epoxy group was observed. Thus, it appears that the progress of the reaction depends considerably on the nucleophilicity of the aromatic compound. In the absence of BF_3_℘OEt_2_, no reaction was observed in any solvent. From this general behavior, we may conclude that the first substitution of **1a** to obtain **6** occurs via a carbocationic intermediate generated with the assistance of the Lewis acid, followed by a nucleophilic attack of an aromatic compound on the cation. In the transformation of **6** into **7**, a reasonable assumption is that the substitution of the hydroxyl group proceeded by an S_N_1- or S_N_2-type mechanism with allylic rearrangement (Scheme 3). 

Generally, the Lewis acid-assisted substitution of the epoxy group is assumed to follow a concerted S_N_2-like mechanism involving a backside attack of a nucleophilic reagent on an epoxide carbon atom [26]. However, such an attack is rather unrealistic, because the fullerene cage occupies the entire side opposite the epoxy ring. The first substitution of the epoxy ring on a fullerene, therefore, would occur with an energetically unfavorable S_N_1-like attack of a nucleophilic reagent on the carbocation generated by the C−O bond cleavage of the epoxy ring. On the other hand, the second substitution for **6** might occur by an S_N_2'-type mechanism, in which the nucleophilic attack on the residual hydroxyl group occurs first, followed by the elimination of -OH on the side with an allylic rearrangement [31]. The fact that no reaction proceeded in the absence of the Lewis acid catalyst suggests that the second substitution occurs by an S_N_1- or S_N_2-type mechanism with allylic rearrangement, where **7** is formed via a carbocationic intermediate with an allylic resonance [32,33,34,35,36], generated with the assistance of BF_3_·OEt_2_. In no case was the formation of 1,2-bis(substituted phenyl)adduct observed, presumably because of the steric hindrance between the two substituents.

### 3.3. Formation of Indolino[2',3':1,2][60]fullerene Derivatives from C_60_O and Aniline Derivatives

**Figure 4:**
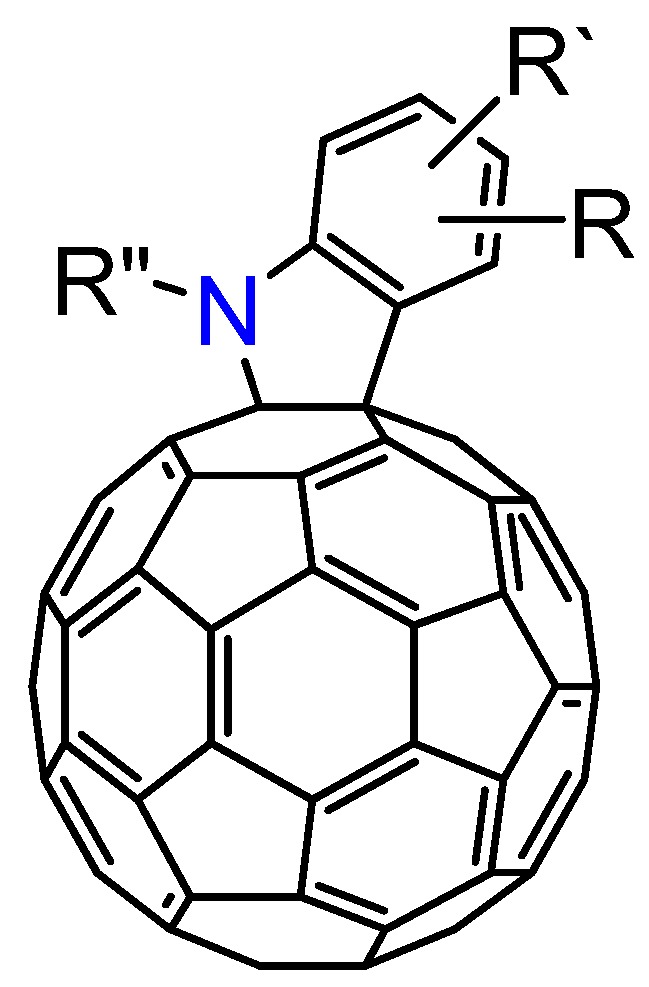
Molecular structure of indolino[2',3':1,2]-1,2-dihydro[60]fullerene derivatives **8**.

As shown in section 3.1, the reactions of C_60_O_x_ with nucleophiles in the presence of acidic compounds have uncovered a means to achieve the regioselective functionalization of various fullerene derivatives. In extending this procedure, we found that a reaction of C_60_O with aniline derivatives in the presence of acidic compounds afforded the indoline-fused fullerene derivatives: indolino[2',3':1,2]-1,2-dihydro[60]fullerene (Figure 4), in moderate to excellent isolated yields (ca. 60–80%) [37]. To the best of our knowledge, only a few cases have thus far been reported on the preparation of indolino[60]fullerene analogues [38,39,40,41,42]. This method enabled access to a wide variety of indolino[60]fullerene derivatives because numerous anilines are commercially available. 

First, we examined the catalytic activities of different types of acidic compound for this reaction by using 4-dodecylaniline as a model compound. The examined acidic compounds were BiCl_3_, BF_3_·OEt_2_, Montmorillonite K10, Sepiolite, TsOH, and Amberlyst 15^®^. The Brønsted acids TsOH and Amberlyst 15^®^ did not exhibit catalytic activities for the reactions, and all of the C_60_O was converted to pristine C_60_. Although the Lewis acid compound; BiCl_3_ gave products with moderate production yields (−50%), BF_3_·OEt_2_ did not give products and only the pristine C_60_ was obtained. The clay compounds, Montmorillonite K10 and Sepiolite exhibited excellent catalytic activities toward the cyclization reactions (Table 5). 

**Table 5:** Catalytic activities of acidic compounds on formation of 5-dodecylindolino[60]fullerene.

Catalyst	Type	Time	Yield of 8a / % ^a^
Montmorillonite K10	clay mineral	6 h	56.7
Sepiolite	clay mineral	6 h	63.8
BiCl_3_	Lewis acid	2 h	50.3
BF_3_·OEt_2_	Lewis acid	2 h	0 (C_60_ was given)
*p*-TsOH	Brønsted acid	over 5 days	0 (C_60_ was given)
Amberlyst 15^®^	Brønsted acid	over 5 days	trace (C_60_ was given)

^a^ Isolated yields, estimated from HPLC areas. Reaction condition: C_60_O 100 mg, 0.136 mmol; 4-dodecylaniline 355 mg, 1.357 mmol; Catalyst ×2,000 wt%, chlorobenzene 100 mL, 100 °C.

The lack of catalytic activity of the Brønsted acid may be attributed to a neutralization process between the Brønsted acid and anilines. For BiCl_3_, the Lewis acidity may be slightly strong; thus, the epoxy oxygen may have been removed from the C_60_ core more quickly than with the nucleophilic addition of anilines. In contrast, BF_3_·OEt_2_ forms a Lewis acid-base complex with aniline. The capped aniline may have lost its nucleophilicity, and thus completely prevented the reaction from proceeding. Cray minerals presumably have moderate Lewis acidity relative to BF_3_, and may thus be appropriate for the reaction.

Subsequently, in order to examine the versatility of this reaction, we carried out the reactions with a wide variety of anilines. The examined anilines and results are summarized in Table 6. Each aniline examined gave the corresponding indolino[60]fullerene derivative in good yield except for the reaction with *N*-Me-*p*-toluidine (Entry **8f**). For **8f**, the steric hindrance of the methyl group on the nitrogen atom might inhibit nucleophilic addition to the carbon atom of the fullerene core.

**Table 6:** Isolated yields of indolino[60]fullerene derivatives.

Entry	Aromatic Amine	5-	*N*-	Yield / % ^a^
**8a**	4-Dodecylaniline	*n*-C_12_H_25_	H	77.7
**8b**	4- *n*-Butylaniline	*n*-C_4_H_9_	H	64.1
**8c**	*p*-Toluidine	Me	H	84.0
**8d**	4-Fluoroaniline	F	H	75.7
**8e**	Aniline	H	H	78.9
**8f**	*N*-Me-*p*-toluidine	Me	Me	29.3

^a^ Isolated yield, estimated from HPLC areas. Reaction conditions: C_60_O 100 mg, 0.136 mmol; aromatic amines 1.357 mmol; Sepiolite 2.0 g; chlorobenzene 100 mL, 100 °C.

**Figure 5:**
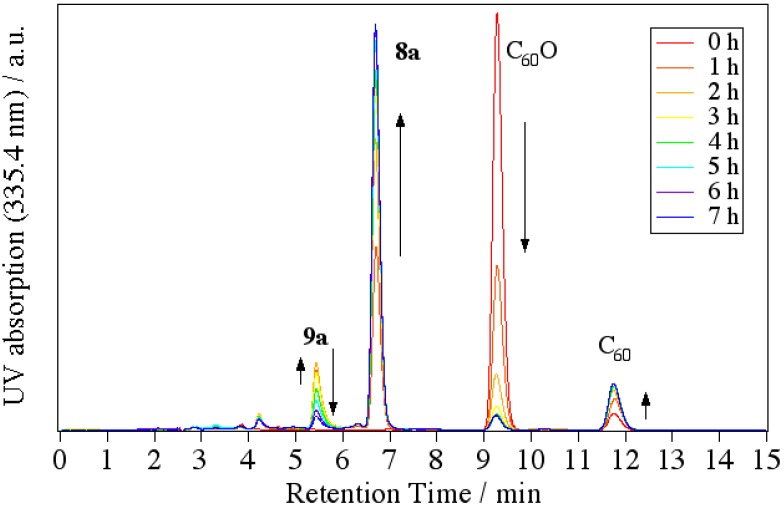
Time course of the HPLC chart for the reaction of C_60_O with 4-dodecylaniline.

Next, we attempted to reveal the reaction mechanism of the formation of indolino[60]fullerene from C_60_O and anilines. By tracing the reaction with an HPLC-MS system, we revealed that the cyclization reaction proceeded via 1,2-anilinoalcohol fullerene derivative. The time course of the HPLC chart and the change in the relative peak area for each compound are shown in Figure 5. 

At the start time, peaks corresponding to C_60_O at 9.3 min and slightly impure C_60_ at 11.8 min appeared. At first, a new peak (**9a**) appeared at 5.4 min in the LC chart, and then, a peak corresponding to **8a** at 6.8 min increased as the peak area of **9a** decreased. Finally, the peaks of the starting C_60_O and **9a** were completely consumed and the peak **8a** and a small peak corresponding to pristine C_60_ as a decomposed compound at 11.8 min were observed. Therefore, we surmised that **9a** was an intermediate of the indolino[60]fullerene derivative. To confirm our assumption, isolated **9a** with the catalyst under similar reaction conditions, whereupon the reaction gave **8a**.

**Scheme 4:**
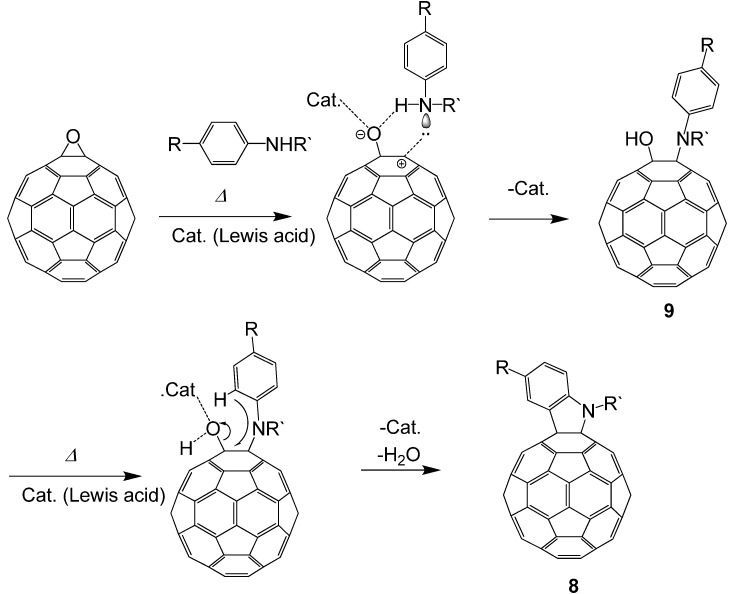
Plausible reaction mechanism of indolino[60]fullerene derivative formation from C_60_O and anilines.

In order to assign each peak, we isolated and characterized compound **9a** by means of IR, APPI-MS, and NMR spectra. In the IR spectrum, an absorption corresponding to O–H stretching was observed at 3,793 cm^–1^. The MS spectrum of **9a** showed a peak at *m*/*z* = 998. The MS of the peak corresponds to the summation of C_60_O (mw = 736) and dodecylaniline (mw = 262). As a result of ^1^H-NMR, we observed in the range of 0.87–2.60 ppm, two doublet peaks of aryl protons corresponding to four aniline protons at 7.19 and 7.61 ppm, and one singlet peak of NH at 5.93 ppm. Taking these results into consideration, we assigned **9a** to a 1-anilino-2-hydroxy[60]fullerene derivative (Scheme 4).

Based on the experimental results, we proposed a plausible mechanism for the formation of indolino[60]fullerene (8) via 1,2-anilinohydroxy fullerene (9) (Scheme 4). At first, the epoxy ring is opened by adding a Lewis acid to the oxygen of the epoxide. Then, nucleophilic addition of anilines to the carbon atom underlying the epoxy oxygen forms a 1,2-anilinohydroxy fullerene derivatives. Consequently, the Lewis acid again adds to the oxygen atom of the hydroxyl group, and a C–C bond forms between the carbon of C_60_ and the *ortho*-position of the anilino group to give the indoline-fused fullerene derivative. 

### 3.4. Formation of Benzo[b]furano[2',3':1,2][60]fullerene Derivatives from C_60_O and Phenols

**Figure 6:**
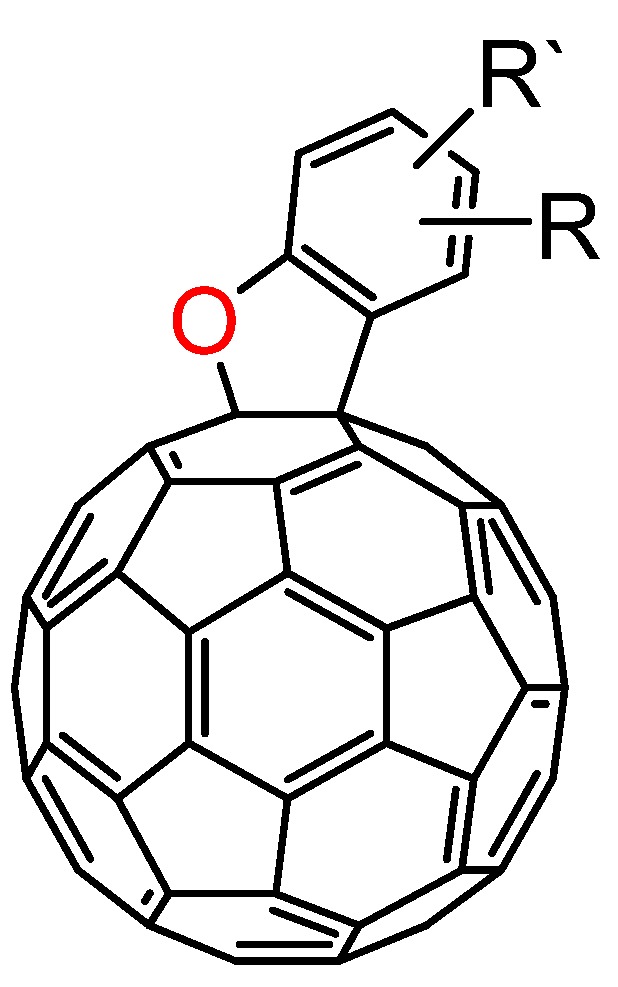
Molecular structure of benzo[*b*]furano[2',3':1,2][60]fullerene derivatives.

A similar nucleophilic addition to the fullerene core and the consequent cascade cyclization reaction with phenols via a 1-hydroxy-2-phenolic fullerene derivative as an intermediate was also observed. This reaction gave benzo[*b*]furano[2',3':1,2][60]fullerene derivatives in good yields as well (Figure 6) [43].

Unlike in the case of anilines, BF_3_·OEt_2_ exhibited superior catalytic activity for the reaction of C_60_O with phenols. The reaction of C_60_O with phenol derivatives in the presence of BF_3_·OEt_2_ was complete within 30 min for nearly all examined phenols. The different catalytic activities of the Lewis acids may be attributed to the Lewis acidity of a catalyst, the Lewis basicity of a nucleophile, or both. For the reaction with anilines, a complexation between BF_3_ and the amine occurs preferentially over the addition of BF_3_ to the epoxy oxygen. Therefore, either BF_3_ or the aniline is completely consumed, and the reaction cannot proceed at all. On the other hand, for benzo[*b*]furano[60]fullerenes, the Lewis basicity of nearly all phenols is weaker than that of anilines. BF_3_ thus effectively catalyzes the reaction of epoxy ring opening, nucleophilic addition, and cyclization.

The production yields of the benzo[*b*]furano[60]fullerene derivatives were subjected to the electron-donating and withdrawing properties of the substituent of the phenols. The isolated yields are summarized in Table 7.

**Table 7:** Isolated yields of benzo[b]furano[60]fullerene derivatives.

Entry	Phenols	Yield / % *^a^*
**10b**	4 *-n*-octylphenol	91.5
**10c**	4- *n*-butylphenol	88.8
**10d**	4-methylphenol	91.5
**10e**	4-methoxyphenol	72.9
**10g**	4-fluorophenol	30.8
**10h**	phenol	41.1
**10i**	3,5-di-methoxyphenol	82.2
**10l**	4-nitrophenol	0

*^a^* Isolated yields, estimated from HPLC areas. Reaction conditions: C_60_O 100 mg, 0.136 mmol; phenols 1.357 mmol; BF_3_·OEt_2_ 37 μL 0.136 mmol; chlorobenzene 100 mL, 70 °C.

**Figure 7:**
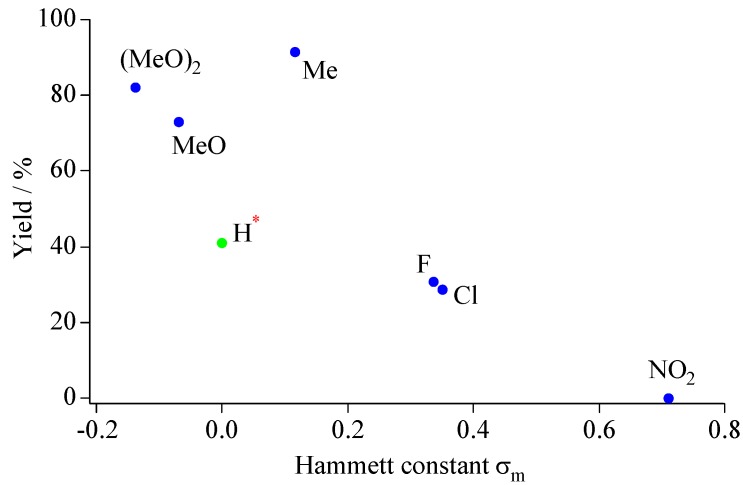
A plot of Hammett constant *vs*. production yields forbenzo[*b*]furano[60]fullerene derivatives.

Unlike the case of anilines, the yields of benzo[*b*]furano[60]fullerene derivatives were strongly influenced by the type of substituent of the phenols. Figure 7 shows a plot of the isolated yields for benzo[*b*]furano[60]fullerene derivatives as a function of the Hammett constant for the 2-position of the precursor 4-substituted phenols [44]. Negative Hammett constants correspond to electron-withdrawing (EW) property, and positive ones correspond to the electron-donating (ED) property. The stronger ED property of the substituent increases the nucleophilicity of the oxygen atom of phenols and the carbon atoms of *ortho*-position of the phenols. In contrast, EW groups reduce the nucleophilicity of the phenolic oxygen and the carbon atoms of *ortho*-position. Therefore, ED functional groups improve the production yields of nucleophilic addition and cascade cyclization reactions. Conversely, 4-nitorophenol could not produce the benzo[*b*]furano[60]fullerene owing to the strong electron-withdrawing property of the nitro group. The low yield with pristine phenol (entry **10h**) is presumably due to the competitive formation of the 1,4-bisadduct (Section 3.2), because the *para*-position of the phenol is not substituted.

## 4. Conclusions

In summary, this review presents an overview of our works on the nature and chemical transformation of fullerene epoxides. The structures of the formed fullerene epoxides, C_60_O_n_, were characterized on the basis of their LC-MS and ^13^C-NMR spectral analyses. The number of carbon peaks and structural symmetry correlations enable the different oxygen addition sites on the fullerene framework to be distinguished. The obtained results show highly regioselective exohedral oxygenation of fullerene with oxidizing agents such as *m*-CPBA and ozone.

A variety of fullerene derivatives are easily prepared from fullerene epoxides, in which the epoxy groups on the fullerene framework are efficiently activated to undergo chemical transformations in the presence of a Lewis acid. We have found several Lewis acid-assisted reactions of fullerene epoxide with various types of nucleophiles. In all of the examined nucleophilic substitution reactions on fullerene epoxides, carbon-oxygen bond cleavage of the epoxy ring occurs with a front side attack of a nucleophile. Moreover, the substitution reaction of the fullerene di-epoxide proceeds while the rearrangement of the two epoxy rings on the fullerene cage is conserved. Fullerene epoxides are 0.39"materials in many industrial fields.
